# Shared Patterns of Brain Functional Connectivity for the Comorbidity between Migraine and Insomnia

**DOI:** 10.3390/biomedicines9101420

**Published:** 2021-10-09

**Authors:** Kun-Hsien Chou, Chen-Yuan Kuo, Chih-Sung Liang, Pei-Lin Lee, Chia-Kuang Tsai, Chia-Lin Tsai, Ming-Hao Huang, Yi-Chih Hsu, Guan-Yu Lin, Yu-Kai Lin, Ching-Po Lin, Fu-Chi Yang

**Affiliations:** 1 Brain Research Center, National Yang Ming Chiao Tung University, Taipei 112, Taiwan; dargonchow@gmail.com (K.-H.C.); chingpolin@gmail.com (C.-P.L.); 2 Institute of Neuroscience, National Yang Ming Chiao Tung University, Taipei 112, Taiwan; lynnlee0226@gmail.com (P.-L.L.); huangminghau@gmail.com (M.-H.H.); 3 Aging and Health Research Center, National Yang Ming Chiao Tung University, Taipei 112, Taiwan; bbmm0208@gmail.com; 4 National Defense Medical Center, Department of Psychiatry, Beitou Branch, Tri-Service General Hospital, Taipei 112, Taiwan; lcsyfw@gmail.com; 5 National Defense Medical Center, Department of Neurology, Tri-Service General Hospital, Taipei 114, Taiwan; jiakuang@yahoo.com.tw (C.-K.T.); jialin0912@gmail.com (C.-L.T.); yuzi711@gmail.com (G.-Y.L.); yukai0907@mail.ndmctsgh.edu.tw (Y.-K.L.); 6 National Defense Medical Center, Graduate Institute of Medical Sciences, Taipei 114, Taiwan; doc31578@gmail.com; 7 National Defense Medical Center, Department of Radiology, Tri-Service General Hospital, Taipei 114, Taiwan; 8 Department of Biomedical Imaging and Radiological Sciences, National Yang Ming Chiao Tung University, Taipei 112, Taiwan

**Keywords:** default mode network, insomnia, migraine, connectivity, somatosensory

## Abstract

Migraine is commonly comorbid with insomnia; both disorders are linked to functional disturbance of the default mode network (DMN). Evidence suggests that DMN could be segregated into multiple subnetworks with specific roles that underline different cognitive processes. However, the relative contributions of DMN subnetworks in the comorbidity of migraine and insomnia remain largely unknown. This study sought to identify altered functional connectivity (FC) profiles of DMN subnetworks in the comorbidity of migraine and insomnia. Direct group comparisons with healthy controls, followed by conjunction analyses, were used to identify shared FC alterations of DMN subnetworks. The shared FC changes of the DMN subnetworks in the migraine and insomnia groups were identified in the dorsomedial prefrontal and posteromedial cortex subnetworks. These shared FC changes were primarily associated with motor and somatosensory systems, and consistently found in patients with comorbid migraine and insomnia. Additionally, the magnitude of FC between the posteromedial cortex and postcentral gyrus correlated with insomnia duration in patients with comorbid migraine and insomnia. Our findings point to specific FC alterations of the DMN subnetwork in migraine and insomnia. The shared patterns of FC disturbance may be associated with the underlying mechanisms of the comorbidity of the two disorders.

## 1. Introduction

Insomnia is one of the most prevalent health complaints worldwide [1]. Approximately 10% of the world population suffers from insomnia [2]. Insomnia, characterized by difficulties in falling asleep, staying asleep, and obtaining refreshing sleep [2], has significant negative impacts on individuals’ health, leading to a severely reduced quality of life including cognitive and affective impairments, work disability, traffic accidents, and a marked increase in health care consumption [3,4,5]. Accumulating evidence indicates that insomnia is closely associated with several neuropsychiatric disorders, especially migraine [6,7,8], a common primary headache disorder that affects about 10–20% of the general population [9]. Migraines are characterized by recurrent lateralized and intense headaches accompanied by nausea, vomiting, and sound and light sensitivity [10]. Poor sleep quality, including insomnia, affects around 30% of migraine patients [11,12]. Insomnia can trigger migraine attacks, and people with chronic migraine are prone to recurrent headaches due to sleep insufficiency [11,12]. Furthermore, previous research suggests that emotional disorders, including depression and anxiety, are associated with migraine and insomnia [13,14]. Individuals with insomnia or migraine may have limitations in daily function and decreased quality of life [15,16], and both insomnia and migraine impose a huge personal and social burden [16,17]. However, the neuronal mechanisms underlying the pathogenesis of insomnia and migraine remain largely unknown.

Functional magnetic resonance imaging (fMRI) is the well-established imaging approach that evaluates functional properties of the human brain non-invasively using the fluctuations of blood oxygen level dependent signals. This approach can be further divided into two main categories: the task-induced fMRI that explores the brain activity in response to task relevant changes and the intrinsic resting state fMRI (rsfMRI) that identifies the intrinsic functional connectivity (FC) of specific cortical regions at rest [18]. Intrinsic FC is defined by statistical associations between distinct brain regions and provides a unique method for studying large-scale functional organization of the human brain [19]. The default mode network (DMN) is the most consistent resting-state network that includes the dorsal medial prefrontal cortex (DMPFC), the posteromedial cortex, and the bilateral temporoparietal junctions. Therefore, DMN is a set of functionally interconnected brain regions that consistently deactivate during the tasks demanding external attention and activate during internal mental processes [20,21].

Previous rsfMRI studies have demonstrated that multiple brain regions within the DMN may act as network hubs for integrating information flow across the entire brain [22,23]. Due to its important role in functional integration of the brain, efforts have been made recently to further assess the role of the DMN in diseases, suggesting that this network can be associated with pathophysiology of neuropsychiatric disorders [24,25]. Prior research has shown FC alternations of the entire DMN in patients with migraine or insomnia, which were associated with pain regulation, emotion processing, and several clinical evaluation readouts [26,27]. Furthermore, poor pain regulation as well as emotional and memory disturbance were common complaints in patients with migraine or insomnia, especially in comorbid patients [4,7,8]. Therefore, functional disturbance of the DMN may be involved in the pathophysiology of migraine and insomnia comorbidity.

Recent evidence suggests that the DMN can be segregated into distinct subnetwork systems underlying different cognitive processes [28,29] and may be associated with pathophysiology of neuropsychiatric disorders [30,31]. The temporoparietal junction is involved in self-referential processing and social cognition [32]. The posterior cingulate cortex has implications in emotional processing, memory retrieval, and attention during task-based activities [33,34]. The DMPFC is engaged in self-referential mental activity and emotional processing [35]. Cognition, memory, attention, and affective impairments are vivid in patients with migraine and insomnia, especially in comorbid patients [3,4,5]. Furthermore, research has shown that functional disruption in posterior cingulate cortex is associated with emotional processing in migraine [36] and sleep quality in insomnia patients [37]. Although the DMN has been widely investigated in patients with migraine and insomnia, little is known about whether there are dissociative functional alternations of DMN subnetworks in both disorders. Furthermore, it remains uncharacterized whether there are any atypical FC patterns of DMN subnetworks associated with the comorbidity of these two disorders. Separating these disease-related DMN subnetworks from a single monolithic DMN may help us to better understand the potential network-based pathophysiology of migraine and insomnia comorbidities.

Therefore, this study sought to explore the mechanisms of migraine and insomnia comorbidity by analyzing the whole-brain voxel-wise intrinsic FC changes of the DMN subnetworks. We hypothesized that FC between DMN subnetworks and a subset of implicated brain areas is consistently altered in migraine and insomnia patients. To this end, (1) direct group comparisons with healthy participants followed by conjunction analyses were used to identify regions of shared FC alterations of DMN subnetworks in both disorders, (2) an additional patient group with comorbid migraine and insomnia was used to validate these shared brain regions, and (3) exploratory association analyses using multiple clinically relevant evaluations were applied to evaluate the potential clinical significance of the identified shared brain regions.

## 2. Materials and Methods

### 2.1. Participant Recruitment and Assessments

The study protocol was approved by the institutional review board of the Tri-Service General Hospital (TSGH). All participants provided informed written consent before enrollment. They were recruited consecutively at the Headache Clinic at the Neurology Department of TSGH. This study included 50 participants diagnosed with migraine (88.0% female), 20 with insomnia (65.0% female), 25 with comorbid migraine and insomnia (88.0% female), and 30 healthy controls (76.6% female).

Primary insomnia was diagnosed using a structured clinical interview according to the DSM-V criteria [38]. Medical and psychiatric disorders were evaluated using structured diagnostic interviews, physical examinations, blood tests, and urine drug testing. Patients with secondary insomnia (e.g., hypersomnia, parasomnia, history of heart disease, stroke, nephritis, or psychiatric diseases, etc.) were excluded during recruitment. Clinical characteristics of all participants diagnosed with primary insomnia, including insomnia duration and insomnia severity, were documented. The Insomnia Severity Index (ISI) is a brief self-reported questionnaire that measures the patient’s perception of insomnia severity [39]. It consists of seven items that evaluate aspects of insomnia symptoms; each ISI item is rated on a scale of 0–4. The total ISI score is divided into four categories: 0–7, no clinically significant insomnia; 8–14, sub-threshold insomnia; 15–21, moderate insomnia; and 22–28, severe insomnia. The Pittsburgh Sleep Quality Index (PSQI) assesses sleep quality over one month. The index combines seven component scores that are added together to produce a final score, and higher scores indicate worse sleep quality. Starting the score of 5, the sleep quality is considered as poor [40].

Migraine was diagnosed according to the third edition of the International Classification of Headache Disorders [41]. Patients with secondary or other concomitant primary headache disorders were excluded. Clinical characteristics of all participants diagnosed with migraine, including migraine duration, frequency, aura symptoms, family history, and headache intensity, were documented. All migraine patients were migraine-free for at least 3 days prior to the examination and were followed-up 3 days after the MRI scanning to ensure that they remained migraine-free to avoid any possible interference from headache pain on the imaging results. No acute migraine attacks occurred during the scanning sessions. No migraine or insomnia patient was treated with any migraine preventive medication, dopaminergic agents, antidepressants, neuroleptics, or hypnotics.

The healthy control group consisted of volunteers recruited through community advertisements, matched for age, sex, and handedness. Exclusion criteria for healthy controls included a prior diagnosis of primary or secondary headache disorders, a family history of insomnia or migraine, a history of other neurological or psychiatric diseases, and any chronic pain condition. Demographic and clinical data including all participants’ sex, age were also documented. To exclude the potential confounders related to depression and anxiety, we evaluated the Hospital Anxiety and Depression Scale (HADS) score [42] in all participants of the study.

### 2.2. MRI Data Acquisitions

All MRI scans were acquired on a 3T Discovery MR750 scanner (General Electric Healthcare, Milwaukee, WI, USA) equipped with an eight-channel head array coil at TSGH. To prevent head motion during image acquisition, cushions were used to constrain the participant’s head position. All structural and functional MRI scans were acquired along the anterior commissure–posterior commissure plane and ensured coverage of the entire brain. Axial T1-weighted anatomical scans were first acquired using the three-dimensional inversion recovery prepared fast spoiled gradient recalled (IR-FSPGR) sequence with the following imaging parameters: repetition time (TR)/echo time (TE)/inversion time = 9.2/3.7/450 ms, flip angle = 12°, slice thickness = 1 mm, image matrix = 256 × 256, field of view (FOV) = 256 × 256 mm, and 172 partitions without an inter-partition gap. These individual T1-weighted images were used for image spatial registration and for segmenting tissue compartments for facilitating rsfMRI image preprocessing. Subsequently, rsfMRI scans were acquired using an axial T2*-weighted gradient-echo echo planar imaging sequence with the following imaging parameters: TR/TE = 2500/30 ms, flip angle = 90°, slice thickness = 3.5 mm, image matrix = 64 × 64, FOV = 222 × 222 mm, 43 interleaved partitions without an inter-partition gap, 200 continuous image volumes with an acquisition time of 8 min and 27 s. Prior to rsfMRI image acquisition, participants were instructed to relax, keeping their eyes closed, to think of nothing in particular, and not to fall asleep. An experienced neuroradiologist visually examined all raw MRI scans prior to image preprocessing to exclude participants with any organic brain disorders or insufficient image quality.

### 2.3. The Analytical Framework of the Present Study

**Figure 1:**
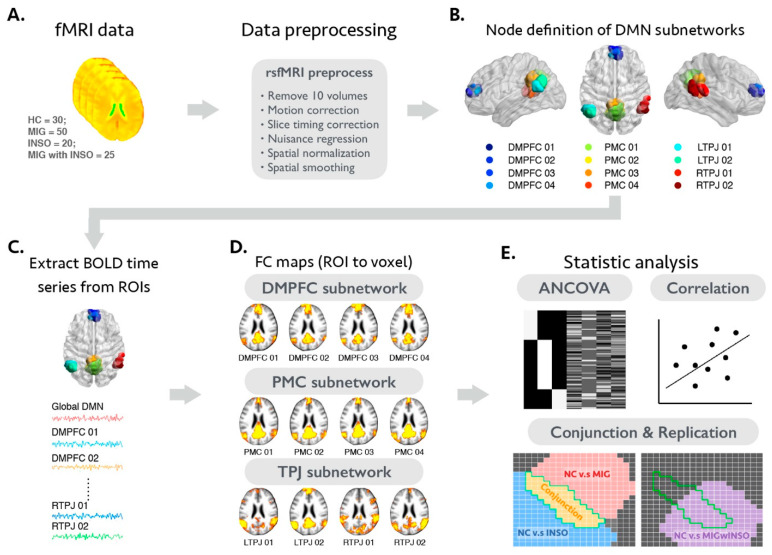
Depiction of the proposed analytical framework in this study. (**A**) A rs-fMRI preprocessing pipeline was used to generate residual rs-fMRI time series data for each individual. (**B**) The definition of the DMN subnodes is based on the previous large-scale connectivity-based parcellation [44]. Different colors represent different nodes of the DMN (DMPFC, 4 nodes; PMC, 4 nodes; and TPJ, 4 nodes). (**C**) The residual time series data were extracted and averaged from the different nodes of the DMN, including global DMN and subnodal DMN. (**D**) Individual voxel-wise FC maps were computed using a Pearson correlation coefficient between the mean time course of each node in the DMN and each voxel in the whole brain and were then transformed to z-value maps. (**E**) Serial statistical analyses were used to identify the shared FC changes of the global DMN and DMN subnetworks in patients with migraine and insomnia and further validated with additional comorbidity group. Abbreviations: BOLD, blood oxygen level dependent; DMN, default mode network; DMPFC, dorsomedial prefrontal cortex; FC, functional connectivity; HC, healthy controls; INSO, insomnia; LTPJ, left temporoparietal junctions; MIG, migraine; MNI, Montreal Neurological Institute; PMC, posteromedial cortex; rs-fMRI, resting-state functional magnetic resonance imaging; ROIs, regions of interest; RTPJ, right temporoparietal junctions.

The proposed analytical framework is summarized in Figure 1. Briefly, after conducting several image preprocessing steps for an individual rsfMRI dataset, we then applied the seed-to-voxel FC analytical approach to estimate the whole brain voxel-wise functional connectivity maps of global and DMN subnetworks for each participant. This approach estimates the potential whole brain voxel-wise functional connectivity profile by analyzing correlations between the average signal fluctuation within pre-defined seed regions and the signal fluctuation within each voxel [43]. Afterwards, corresponding statistical analyses were performed to identity the shared FC alternations between patients with migraine and insomnia and further validated with the additional comorbidity patient group. The detailed analytic steps are summarized below.

### 2.4. Image Preprocessing of Individual rsfMRI Dataset

All neuroimaging data were preprocessed using the combination of three neuroimaging software packages: Analysis of Functional Neuroimaging software package (AFNI, version 20.3.03, https://afni.nimh.nih.gov/, RRID:SCR_005927, accessed on 7 September 2021), Functional Magnetic Resonance Imaging of the Brain Software Library (FSL, version 5.0.10, http://www.fmrib.ox.au.uk/fsl, RRID:SCR_002823, accessed on 7 September 2021), and Statistical Parametric Mapping (SPM12, version 7487, https://www.fil.ion.ucl.ac.uk/spm/, RRID:SCR_007037, accessed on 7 September 2021). The detailed preprocessing pipeline is as follows. First, the first ten volumes of individual rsfMRI dataset were discarded for signal equilibrium. Before any spatiotemporal processing procedure, individual head movement during rsfMRI data acquisition was corrected and corresponding motion parameters (rotation and translation profiles of XYZ axis) were estimated using rigid-body registration with default settings (mcflirt). The slice timing correction was then applied for correcting the temporal misalignment during image acquisition (slicetimer). Using the Brain Extraction Tool, the skull-stripped version of the individual preprocessed rsfMRI data was generated. Additionally, the spike artifact and the polynomial trend of fMRI time series data were also removed (3dDespike and 3dDetrend). To further minimize the potential influence of non-neural signal, band-pass temporal filtering (0.01–0.08 Hz) and nuisance signal regression procedure were conducted simultaneously using command-line tool 3dBandpass. Twenty-six nuisance signal regressors, including (1) six head motion profiles along with the corresponding temporal derivatives and quadratic terms (also termed as Friston 24-parameter model [45]); and (2) mean rsfMRI time-series signal from both white matter (WM) and cerebrospinal fluid (CSF) areas were constructed in native rsfMRI space for each individual. Individual mean time-series data of WM and CSF area were calculated separately within the corresponding WM and CSF masks, which were generated using the segmentation module of SPM12. To avoid suppressing meaningful neural activity and artificially inducing the anti-correlation functional connectivity profile, the mean global rsfMRI signal was not used as a nuisance signal in the present study [46]. Afterwards, all the individual preprocessed rsfMRI time series data were projected into the standard Montreal Neurological Institute (MNI) space with a two-stage registration approach [47]. More specifically, we first used command-line tools 3dAllineate (linear registration) and 3dQwarp (non-linear registration) to estimate all the spatial transformation information between individual native rsfMRI space, native T1 space, and the standard MNI space. All the relevant spatial transformation information was then contracted into a single spatial warping file using the command-line tool 3dNwarpCat and then applied to project individual preprocessed rsfMRI datasets into the MNI space (3dNwarpApply). Finally, all the individual MNI-space preprocessed rsfMRI datasets were spatially smoothed with a 6 mm full-width at half-maximum Gaussian kernel and then served as inputs for evaluating whole-brain voxel-wise functional connectivity profiles of global and DMN subnetworks.

### 2.5. Selection Criteria and Analysis of Motion-Related Issues of the rsfMRI Dataset

Several previous studies have indicated that the degree of individual head motion is a key confounding factor for estimating functional connectivity profile of the human brain [48]. To minimize the potential influence of this issue, additional selection criteria and relevant statistical analyses were also conducted for the current study. First, any participant who demonstrated a maximum displacement of 2 mm or rotation of 2° in any directions during rsfMRI data acquisition was excluded. Second, we excluded participants with mean framewise displacement (FD) > 0.2 mm or exceeding 40% of image volumes after censoring all time points with FD > 0.2 mm. The FD was defined as the sum of the absolute values of the derivatives of six rotational and translational realignment parameters at each timepoint. These aggregated indices represented the profile of head micromovement during rsfMRI acquisition for each individual [43]. More specifically, we used the command-line tool fsl_motion_outliers to calculate individual FD profiles. Under these two criteria, 5 participants with migraine, 4 with insomnia, 2 with comorbid migraine and 5 healthy controls were excluded. In the remaining dataset, there were no statistically significant group differences in mean FD (healthy controls = 0.073 ± 0.02; patients with migraine = 0.074 ± 0.02; patients with insomnia = 0.072 ± 0.02; and patients with migraine and insomnia = 0.075 ± 0.02; *p*-value = 0.512). Additionally, the mean FD of each participant served as the covariate of no interest for the following group-level statistical analyses.

### 2.6. Functional Connectivity Analysis and Statistical Criteria of Voxel-Wise Statistical Analyses

To investigate the FC alterations of global and subnodal DMN between study groups, whole brain seed-to-voxel FC analyses with appropriate statistical analyses were conducted. We used the well-established DMN atlas, which is constructed according to whole-brain connectivity profiles, to identify the set of seeds of interests in the current study. It is composed of four distinct brain areas, including the DMPFC (4 nodes), the posteromedial cortex (4 nodes), and the bilateral temporoparietal junctions (4 nodes) [44]. The mean time course of each seed region was first extracted, and these 12 time-courses were then averaged and represent the overall time-series signal of global DMN. Subsequently, we calculated Pearson’s correlation coefficient between these mean time-courses to all voxels in the whole brain for each participant. Using Fisher’s r-to-z transformation, the correlation coefficient maps were converted into z-value maps and were used for further group-level analyses. For all the following voxel-wise between-group statistical analyses, the image-based multiple-comparison correction was performed using 3dClusterSim. The significance level was set at a cluster-level family-wise error (FWE) rate-corrected *p*-value < 0.05, which was equal to a combination threshold of an initial voxel-level *p*-value < 0.005 with a minimum cluster size of 111 voxels. For data reusability and transparency, all the unthresholded voxel-wise statistical maps are available at the NeuroVault website (https://neurovault.org/collections/9467/, accessed on 7 September 2021). The detailed settings of each statistical model are listed below.

### 2.7. Statistical Analyses

#### 2.7.1. Demographic Data, Clinical Evaluations, and Motion Profiles of rsfMRI Dataset

All statistical analyses of demographic variables, clinical evaluations, and mean FD were performed using the Statistical Package for Social Sciences (SPSS, V.20, Chicago, IL, USA). Analysis of variance tests and Pearson’s chi-square test were used to compare continuous (age) and categorical (sex and aura status) data between study groups. In addition, analysis of covariance (ANCOVA) test was performed to compare multiple clinical evaluations (migraine duration, insomnia duration, migraine frequency, total ISI score, total PSQI score, and HADS score) and mean FD between groups, with age and sex as covariates. The threshold of statistical significance was set at *p*-value < 0.05 for all statistical tests.

#### 2.7.2. Analysis of FC Differences between Patients with Migraine, Patients with Insomnia, and Healthy Controls to Identify Shared and Distinct Functional Alterations

The two-stage statistical scheme was used to identify the shared functional alterations of DMN between healthy controls and patients with migraine or insomnia [49]. More specifically, we first assessed FC changes of DMN between healthy controls and patient groups (healthy controls vs. migraine and healthy controls vs. insomnia) using an ANCOVA statistical design in SPM12 with age, sex, mean FD, and HADS as nuisance variables. Afterwards, we performed a conjunction analysis that searched the intersection of the whole-brain voxel-wise FWE-corrected *p*-maps to identify the shared FC alterations of DMN in patients with migraine and those with insomnia. Moreover, the direct disease group comparison (migraine vs. insomnia) was also performed to identify the distinct FC changes of DMN between these two patient groups.

#### 2.7.3. Validation Analysis of Shared Functional Alterations in an Additional Comorbid Migraine and Insomnia Group

To evaluate the shared functional signatures of DMN, in which it was identified in the previous section whether they were consistently found in the additional comorbid patient group, we first applied the same ANCOVA statistical model to investigate the whole-brain FC alterations of DMN between healthy controls and patients with comorbid migraine and insomnia. Subsequently, we spatially overlapped these thresholded voxel-wise statistical result maps with previous results of the DMN conjunction analysis to evaluate the correspondence between these two analyses.

#### 2.7.4. Relationship between Regional FC Changes and Clinical Evaluations

To further investigate the potential relationship between regions with shared FC alterations and clinical evaluations in patients with comorbid migraine and insomnia, the regional FC magnitudes were first extracted and averaged from the identified clusters of conjunction analysis. The exploratory partial Pearson correlation analyses were then used to examine the associations between regional FC magnitude and clinical evaluations (including migraine duration, migraine frequency, insomnia duration, ISI total score, and PSQI total score), with age, sex, mean FD, and HADS serving as nuisance variables. The significance threshold for the partial Pearson correlation analyses was set at uncorrected *p*-value < 0.05.

## 3. Results

### 3.1. Demographic and Clinical Characteristics of Study Participants

Major clinical characteristics of participants are presented in Table 1. All groups were matched for age, sex, and handedness. There were significant differences in the headache frequency (*p*-value = 0.003) and insomnia severity (ISI total score) between patient groups (*p*-value < 0.001). The insomnia severity was greater in the insomnia group compared to healthy controls (*p*-value = 0.001) and the migraine group (*p*-value = 0.001), and greater in the migraine and insomnia group than in healthy controls (*p*-value = 0.032) and migraine group (*p*-value = 0.023). Pittsburgh Sleep Quality Index scores were greater in the insomnia group than in the healthy controls (*p*-value < 0.001) and the migraine group (*p*-value < 0.001), and greater in the migraine and insomnia group than in healthy controls *(p*-value < 0.001) and the migraine group (*p*-value < 0.001).

### 3.2. Differences in the FC Magnitude of the Global DMN and Subnodal DMN between Healthy Controls and Patients with Migraine or Insomnia

#### 3.2.1. FC Changes of the Global DMN in Patients with Migraine or Insomnia

Increased global DMN FC magnitude was observed in the left precentral gyrus, left postcentral gyrus, and right cerebellum in patients with migraine or insomnia compared with healthy controls. No anatomical area with decreased global DMN FC magnitude was found in patients with migraine or insomnia when compared with healthy controls (FWE-corrected *p*-value < 0.05; Supplementary Table S1 and Supplementary Figure S1).

#### 3.2.2. FC Changes of the Subnodal DMN in Patients with Migraine or Insomnia

Detailed FC changes of each subnodal DMN in patients with migraine or insomnia relative to healthy controls are shown in Supplementary Table S1 and Supplementary Figure S1 (FWE-corrected *p*-value < 0.05).

#### 3.2.3. FC Changes of the Global and Subnodal DMN between Patients with Migraine or Insomnia

Detailed FC differences of the global DMN and subnodal DMN between patients with migraine and insomnia are shown in Supplementary Table S1 and Supplementary Figure S2 (FWE-corrected *p*-value < 0.05).

### 3.3. FC Alterations of the Global and Subnodal DMN Common to Migraine and Insomnia

**Figure 2:**
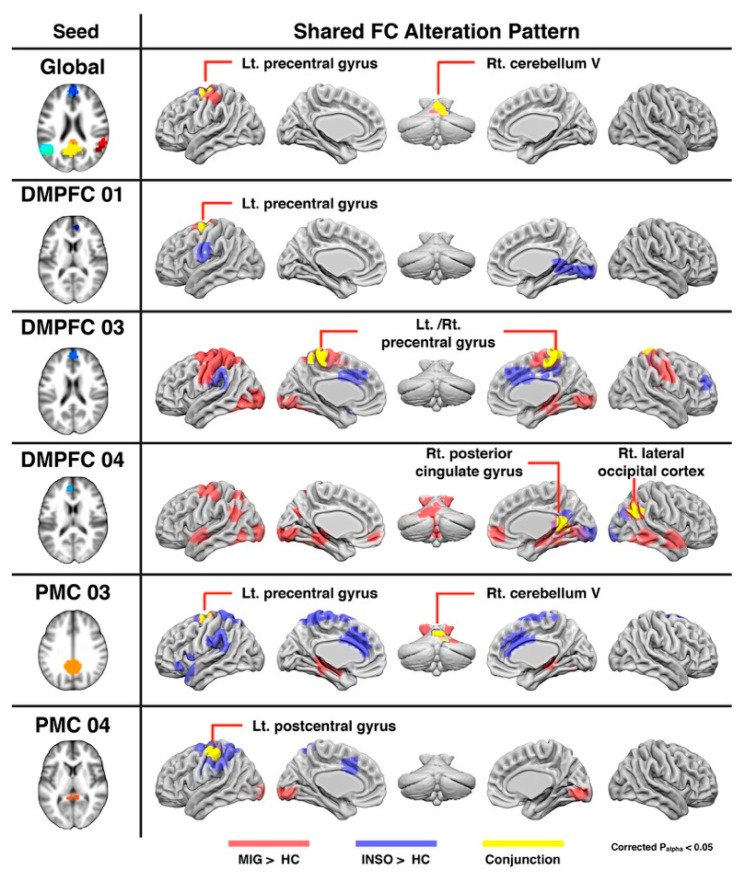
Anatomical regions with shared FC changes of the global DMN and subnodal DMN in the clinical groups (migraine and insomnia) compared with healthy controls. Red regions reflect significantly increased FC in the patients with migraine. Blue regions show significantly increased FC in the patients with insomnia. Yellow regions show the shared FC alterations in both migraine and insomnia patients. Abbreviations: DMPFC, dorsomedial prefrontal cortex; FC, functional connectivity; HC, healthy controls; INSO, insomnia; Lt, left; MIG, migraine; PMC, posteromedial cortex; Rt, right.

First, the conjunction analysis of the global DMN revealed that the left precentral gyrus and right cerebellum showed increased global DMN FC magnitude in both disorders compared with healthy controls (FWE-corrected *p*-value < 0.05; Table 2 and Figure 2). Further conjunction analyses of subnodal DMN further demonstrated only part of the DMN networks displayed common FC alterations in patients with migraine and insomnia. More specifically, the conjunction analyses of the subnodal DMPFC-01 area showed that the FC magnitude of the left precentral gyrus significantly changed in patients with both diseases. The conjunction analyses of subnodal DMPFC-03 area showed that the FC magnitude of the bilateral precentral gyrus significantly changed in patients with both diseases. The conjunction analyses of the subnodal DMPFC-04 area showed that the FC magnitude of the right posterior cingulate gyrus and right lateral occipital cortex significantly changed in patients with both diseases. The conjunction analyses of the subnodal posteromedial cortex-03 area showed that the FC magnitude of the left precentral gyrus and right cerebellum significantly changed in patients with both diseases. Finally, the conjunction analyses of the subnodal posteromedial cortex-04 area showed that the FC magnitude of the left postcentral gyrus significantly changed in patients with both diseases (FWE-corrected *p*-value < 0.05; Table 2 and Figure 2).

### 3.4. Altered FC Patterns of the Global and Subnodal DMN in Patients with Comorbid Migraine and Insomnia

**Figure 3:**
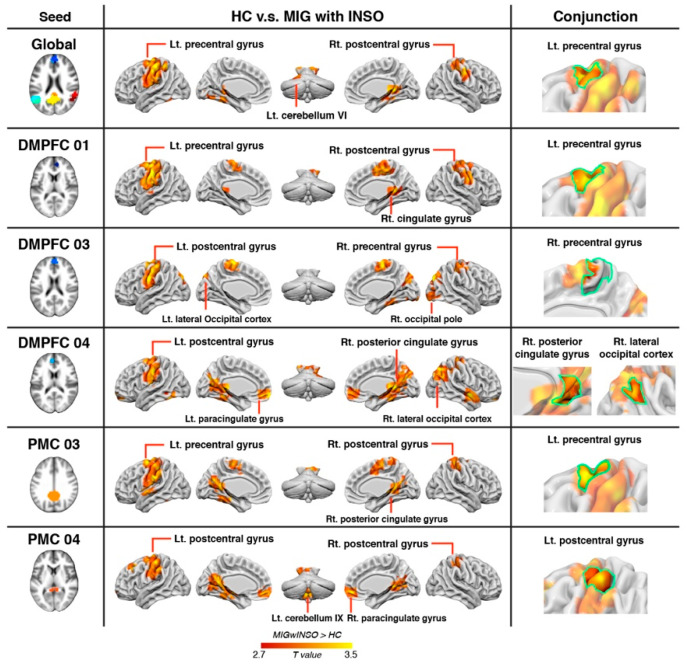
Anatomical regions with shared FC differences of the global DMN and subnodal DMN in patients with comorbid migraine and insomnia (compared with healthy controls). Hot (red) regions show significantly increased FC in patients with comorbid migraine and insomnia. The green boundary area represents the shared FC alterations in migraine and insomnia patients. Abbreviations: DMPFC, dorsomedial prefrontal cortex; FC, functional connectivity; HC, healthy controls; INSO, insomnia; Lt, left; MIG, migraine; PMC, posteromedial cortex; Rt, right.

The global and subnodal FC changes of the DMN in patients with comorbid migraine and insomnia compared with healthy controls are shown in Supplementary Table S2 and Supplementary Figure S3 (FWE-corrected *p*-value < 0.05). Conjunction analyses further demonstrated that increased global and subnodal DMN FC magnitudes were consistently found in the bilateral precentral gyrus, right posterior cingulate gyrus, right lateral occipital cortex, and left postcentral gyrus in patients with comorbid migraine and insomnia relative to the healthy control group (Table 3 and Figure 3).

### 3.5. Potential Clinical Significance of Global and Subnodal DMN FC Changes in the Comorbidity Group

The exploratory partial Pearson correlation analysis revealed that the FC magnitude between the posteromedial cortex-04 area and the left postcentral gyrus correlated with insomnia duration in patients with comorbid migraine and insomnia (*r* = 0.513, uncorrected *p*-value = 0.021).

## 4. Discussion

This study found that patients with migraines or insomnia showed FC changes of the global DMN in the precentral gyrus, postcentral gyrus, and cerebellum compared with healthy controls. FC changes of all the subnodal DMN were primarily observed in the brain motor and somatosensory systems of patients with migraines or insomnia. The conjunction analysis further demonstrated shared FC changes in the DMPFC and posteromedial cortex sub-networks in the precentral gyrus, cerebellum, posterior cingulate gyrus, lateral occipital cortex, and posterior central gyrus of patients with migraines and insomnia. The shared patterns of the subnodal FC alteration areas in both groups were consistently found in the additional comorbid migraine and insomnia group. Moreover, the FC between the posteromedial cortex and the postcentral gyrus correlated with insomnia duration in patients with comorbid migraine and insomnia.

Comorbidities of migraine and insomnia may be related to chronic painful disorders and other irritating symptoms; the resulting physical or psychological pressure indirectly leads to abnormalities in relevant cognitive behaviors or brain functional networks [50]. In addition, chronic pain input may provoke hypersensitivity to pain, resulting in altered brain function and activity [51,52]. In addition, chronic pain intensifies over time, sensitizing brain function, which in turn induces functional changes. Moreover, previous studies have demonstrated that chronic pain is accompanied by a significant increase in FC of the DMN in patients with chronic insomnia [53]. In this study, compared with healthy controls, an increased magnitude of the DMN FC was clearly seen in patients with migraine or insomnia as well as patients with both migraine and insomnia. Taken together, the DMN may be considered as a potential network marker for exploring the common pathogenesis of these two disorders.

The DMN is known to be responsible for sensory integration and adjustment of pain cognition and attention [21]. Previous research reported that the FC magnitude within the DMN is reduced, but that the FC between the DMN and outer brain regions is increased in migraine and insomnia patients [26,54,55,56,57]. This phenomenon may be due to a compensatory effect, in which the original DMN’s function is decreased. Our results also showed an increased FC magnitude between DMPFC/posteromedial cortex and brain regions outside the traditional DMN in migraine and insomnia patients. The FC changes in the DMN subnetwork, especially in the DMPFC and posteromedial cortex sub-regions, might be associated with dysfunctional emotional processing and pain regulation in migraine and insomnia patients [58,59].

The conjunction analysis further demonstrated that the DMPFC and posteromedial cortex sub-networks had shared FC changes with the precentral gyrus, posterior central gyrus, cerebellum, posterior cingulate gyrus, and lateral occipital cortex. In addition to participating in pain perception, the posterior cingulate gyrus is found to be dysfunctional in patients with insomnia [60,61,62]. In this study, we demonstrated that the FC between the DMPFC and posterior cingulate gyrus was increased in migraine and insomnia patients compared to healthy controls. This increased FC magnitude may be associated with a compensatory effect in relation to abnormal pain perception and insomnia. Additionally, the posteromedial cortex and its key brain regions, the anterior wedges (precuneus), are considered to be essential elements of consciousness. These areas are responsible for many higher-order cognitive functions, including contextual memory extraction and self-processing, self-attention transfer, and regulating the sensitivity to pain [63,64]. Therefore, the brain regions that are functionally connected externally from the posterior medial cortex might be potential targets for exploring the mechanisms of pain and sleep regulation.

The precentral gyrus belongs to the sensorimotor cortex and is involved in pain management, especially in chronic pain disorders [65,66]. Migraine patients have decreased gray matter volume in the precentral gyrus, and the precentral gyrus is over-activated during headache attacks, probably due to abnormal cerebral blood flow. The resulting ischemia leads to local structural and neuropathic changes in migraine [67]. Notably, repetitive transcranial magnetic stimulation in the precentral gyrus can effectively suppress pain, indicating that the precentral gyrus may be the focal brain area for chronic pain treatment [68]. In addition, an altered regional homogenous functional activity and neural low-frequency amplitude of the precentral gyrus have also been identified in insomnia patients. Furthermore, previous research demonstrated an atypical FC profile between the precentral gyrus and amygdala [60,69,70]. Patients with insomnia often experience anxiety and depression due to abnormal sleep history, and these clinical symptoms are significantly associated with the central cingulate cortex and the precentral gyrus [69]. Therefore, abnormal changes in the function of the precentral gyrus may help to understand the relationship between the possible neural mechanisms of insomnia and clinical features. In this study, we found an increased FC magnitude between the posteromedial cortex and the precentral gyrus in insomnia and migraine patients, which may be due to the increased pain regulation and sleep disturbances required by the patient.

The cerebellum is an important anatomical region involved in trigeminal nociception and pain modulation [71]. Our results demonstrating an increased FC magnitude between the posteromedial cortex and the cerebellum may therefore be due to altered pain modulation in patients with migraine. The posterior central gyrus has also been suggested to be involved in the trigemino-thalamo-cortical nociceptive pathway, which is implicated in migraine pathophysiology [72]. Furthermore, brain activity in the cerebellum and posterior central gyrus were both reported to be altered in patients with insomnia [73]. Prior research also showed altered FC in the posterior central gyrus and cerebellum, pointing to aberrant sensory processing in patients with insomnia [74]. Additionally, altered FC between the cerebellum and posterior central gyrus has been reported in patients with migraine [75]. Collectively, our results showing an altered DMN FC magnitude in the cerebellum and posterior central gyrus area common to both disease groups may suggest that aberrant motor control and sensory processing are associated with the pathophysiological mechanisms underlying the comorbidity between migraine and insomnia.

In this study, we observed a positive association of the FC magnitude between the posteromedial cortex and the postcentral gyrus with insomnia duration in patients with comorbid migraine and insomnia. Consistently, previous studies have suggested that the FC between the insula and several sensorimotor regions, including the postcentral gyrus, is associated with the insomnia index, pointing to a disturbance in salience processing in patients with insomnia [74]. Therefore, the FC between the posteromedial cortex and the postcentral gyrus might be a potential imaging signature for patients with comorbid migraine and insomnia. In our study, we suggest that aberrant global and subnodal DMN FC is common in both diseases, further suggesting that the aberrant sensory processing and motor control pathways in these specific vulnerable regions may be involved in the pathophysiology of comorbidity between migraine and insomnia.

Our findings may indicate a dysfunction of the glymphatic system in migraine and insomnia. The glymphatic pathways comprise a brain-wide network of perivascular spaces that supports the clearance of metabolic products [76]. The glymphatic system is active primarily during sleep, and its impairment has recently been linked to neurological diseases, such as Alzheimer’s disease, stroke, sleep disturbance, and migraine [77,78,79]. Therefore, a dysfunctional glymphatic system may contribute to the comorbidity of migraine and insomnia. The clinical visualization and quantification of the glymphatic processes are still emerging [80,81]. Time-resolved MRI using a human CSF tracer demonstrated that the contrast agent spreads centripetally from cortex to deeper brain regions, including the cerebral cortex, white matter, limbic system, and cerebellar cortex, suggesting that modulating the glymphatic function can facilitate the delivery of intrathecal compounds [81]. The above-mentioned brain areas are components of the DMN, and further research is needed to determine the potential relationship between the DMN alterations and glymphatic impairments in the comorbidity between migraine and insomnia.

This study has some limitations. First, we used a cross-sectional group comparison design, and the causality of the comorbidity relationship can therefore not be assessed. Second, the DMN template used in this study was constructed using meta-analytic connectivity modeling and a connectivity parcellation approach from a huge amount of collected rsfMRI and positron emission tomography experiments in healthy participants. However, recent research has revealed that a certain degree of variability in functional network organization may exist across individuals [82,83]. Hence, future studies incorporating an individual-wise subnetwork mapping approach will be needed to provide more detailed information on DMN subnetworks in the context of the pathophysiological underpinnings of migraine and insomnia. Third, polysomnography can be used in future studies to select insomnia patients more carefully. This diagnostic test reveals sleep-related breathing disorders, narcolepsy, sleep-related movement disorders, and certain parasomnias [84]. In our study, none of the participants underwent polysomnography. This assessment is not routinely indicated for diagnosing and evaluating insomnia, but it can be used to exclude participants with other sleep-related disorders that affect breathing or movement [84]. Further research using polysomnography will provide objective measurements for selecting the patients with insomnia more accurately. Finally, in this study, the number of male participants was only six to seven in each group (control, migraine, insomnia), and only three in the comorbid group. Therefore, male participants were under-represented, making further statical analysis of sex differences difficult. Since sex differences may play a role in the comorbidity between migraine and insomnia, further research, including more male participants, should be considered.

## 5. Conclusions

In conclusion, this study identified global and subnodal DMN FC alterations in patients with migraine, insomnia, and comorbid migraine and insomnia. Patients with migraine and insomnia displayed a shared regional pattern of FC changes, which mainly affect the brain motor and sensorimotor systems. Additionally, these shared FC alternations were consistently found in patients with comorbid migraine and insomnia, and the FC between the posteromedial cortex and the postcentral gyrus was associated with insomnia duration. Our findings shed new light on the underlying mechanisms of the comorbidity between migraine and insomnia.

## Figures and Tables

**Table 1:** Demographic variables and clinical characteristics of study participants.

Demographic Variables	HC (*n* = 30)	MIG (*n* = 50)	INSO (*n* = 20)	MIG with INSO (*n* = 25)	*p* Value
Age (years)	41.47 ± 10.41	40.14 ± 8.83	40.15 ± 10.25	41.9 ± 11.63	0.863 ^a^
Sex (male/female)	7/23	6/44	7/13	3/22	0.102 ^b^
Framewise Displacement	0.073 ± 0.02	0.074 ± 0.02	0.072 ± 0.02	0.076 ± 0.02	0.512 ^e^
Aura /no Aura	-	6/44	-	7/18	0.084 ^c^
Migraine duration (years)	-	15.63 ± 10.55	-	15.00 ± 10.68	0.622 ^d^
Migraine freq. (days/month)	-	7.38 ± 5.58	-	12.70 ± 9.38	0.003 ^d^
Insomnia duration (month)	-	-	21.32 ± 22.88	15.54 ± 10.31	0.313 ^d^
ISI total score (0–28)	7.03 ± 4.45	6.80 ± 4.79	15.95 ± 3.65	16.36 ± 4.33	<0.001 ^e^
PSQI total score (0–21)	7.19 ± 3.43	8.00 ± 3.61	11.05 ± 3.52	11.00 ± 3.51	<0.001 ^e^
HADS score (0–42)	9.83 ± 4.98	12.18 ± 7.19	13.45 ± 8.66	15.97 ± 7.74	0.018 ^e^

Notes: healthy controls and patients with migraine, insomnia, or comorbid migraine and insomnia were all right-handed. ^a^ Four-group analysis of variance test. ^b^ Four-group chi-square test. ^c^ Two-group chi-square test. ^d^ Two-group analysis of covariance adjusted for age and sex. ^e^ Four-group analysis of covariance adjusted for age and sex. Abbreviations: freq, frequency; HADS, Hospital Anxiety and Depression Scale score; HC, healthy controls; INSO, insomnia; ISI, Insomnia severity index; MIG, migraine; PSQI, Pittsburgh Sleep Quality Index.

**Table 2:** Anatomical regions with shared FC changes in migraine patients and insomnia patients compared with healthy controls.

	MNI Coordinates			Cluster Size	Anatomical Region	Regional FC (Mean ± SD)		
	x	y	z			HC	MIG	INSO
*Global DMN*	−24	−10	68	68	Lt. Precentral Gyrus	0.025 ± 0.185	0.175 ± 0.152	0.215 ± 0.174
	6	−79	−6	100	Rt. Cerebellum V	0.251 ± 0.147	0.403 ± 0.139	0.451 ± 0.134
*DMPFC 01*	−23	−9	67	59	Lt. Precentral Gyrus	−0.031 ± 0.154	0.111 ± 0.151	0.151 ± 0.167
*DMPFC 03*	−4	−42	65	37	Lt. Precentral Gyrus	0.072 ± 0.141	0.208 ± 0.146	0.264 ± 0.159
	7	−39	71	24	Rt. Precentral Gyrus	0.063 ± 0.140	0.215 ± 0.151	0.236 ± 0.205
*DMPFC 04*	8	−46	12	30	Rt. Cingulate Gyrus, posterior division	0.248 ± 0.144	0.374 ± 0.165	0.424 ± 0.148
	55	−62	32	62	Rt. Lateral Occipital Cortex, superior division	0.218 ± 0.160	0.365 ± 0.180	0.415 ± 0.135
*PMC 03*	−27	−10	69	40	Lt. Precentral Gyrus	−0.033 ± 0.152	0.109 ± 0.187	0.166 ± 0.193
	6	−77	−6	333	Rt. Cerebellum V	0.177 ± 0.167	0.336 ± 0.135	0.391 ± 0.149
*PMC 04*	−45	−22	57	85	Lt. Postcentral Gyrus	0.153 ± 0.181	0.294 ± 0.177	0.327 ± 0.221

Peak of group differences in FC magnitude of the global DMN and subnodal DMN with a threshold of FWE-corrected *p*-value < 0.05. Abbreviations: DMN, default mode network; DMPFC, dorsomedial prefrontal cortex; FC, functional connectivity; HC, healthy controls; INSO, insomnia; Lt, left; MIG, migraine; MNI, Montreal Neurological Institute; PMC, posteromedial cortex; Rt, right; SD, standard deviation.

**Table 3:** Anatomical regions with shared significant FC changes in patients with comorbid migraine and insomnia compared with healthy controls.

	MNI Coordinates			Cluster Size	Maximum T Value	Anatomical Region	Regional FC (Mean ± SD)	
	x	y	z				HC	MIGwINSO
*Global DMN*	−22	−16	76	391	5.61	Lt. Precentral Gyrus	0.063 ± 0.141	0.253 ± 0.134
*DMPFC 01*	−22	−14	76	1371	5.96	Lt. Precentral Gyrus	0.039 ± 0.111	0.179 ± 0.117
*DMPFC 03*	4	−14	68	363	4.18	Rt. Precentral Gyrus	0.040 ± 0.106	0.197 ± 0.134
*DMPFC 04*	9	−41	7	1664	4.67	Rt. Cingulate Gyrus, posterior division	0.161 ± 0.102	0.324 ± 0.099
	38	−61	39	465	5.05	Rt. Lateral Occipital Cortex, superior division	0.079 ± 0.086	0.234 ± 0.118
*PMC 03*	−25	−14	74	340	4.88	Lt. Precentral Gyrus	−0.015 ± 0.104	0.154 ± 0.086
*PMC 04*	−57	−22	44	484	4.83	Lt. Postcentral Gyrus	0.106 ± 0.147	0.291 ± 0.186

Peak of group differences in FC magnitude of the global DMN and subnodal DMN with a threshold of FWE-corrected *p*-value < 0.05. Abbreviations: DMN, default mode network; DMPFC, dorsomedial prefrontal cortex; FC, functional connectivity; HC, healthy controls; Lt, left; MIGwINSO, migraine with insomnia; MNI, Montreal Neurological Institute; PMC, posteromedial cortex; Rt, right; SD, standard deviation.

## Data Availability

All data are available from the corresponding author upon request.

## Supplementary Materials

The following are available online at https://www.mdpi.com/article/10.3390/biomedicines9101420/s1, Figure S1: FC differences of global DMN and subnode DMN between patients with migraine and insomnia. Figure S2: FC differences in global DMN and subnode DMN among healthy controls and migraine and insomnia patients. Figure S3: FC differences in global DMN and subnode DMN between healthy controls and patients with comorbid migraine and insomnia. Table S1: Anatomical regions with significant FC changes in migraine patients, insomnia patients compared with healthy controls. Table S2: Anatomical regions with significant FC changes in patients with comorbid migraine and insomnia compared with healthy controls.

## Abbreviations

AFNI: Analysis of Functional Neuroimaging software package; ANCOVA: analysis of covariance; CSF: cerebrospinal fluid; DMN: default mode network; DMPFC: dorsal medial prefrontal cortex; FC: functional connectivity; FWE: family-wise error; FSL: Functional Magnetic Resonance Imaging of the Brain Software Library; HADS: Hospital Anxiety and Depression Scale; ISI: Insomnia Severity Index; MNI: Montreal Neurological Institute; rsfMRI: resting-state functional MRI; SPM: Statistical Parametric Mapping; WM: white matter.

## References

[1] Morin C.M., Benca R. (2012). Chronic insomnia. Lancet.

[2] Ohayon M.M. (2002). Epidemiology of insomnia: What we know and what we still need to learn. Sleep Med. Rev..

[3] Léger D., Partinen M., Hirshkowitz M., Chokroverty S., Touchette E., Hedner J. (2010). Daytime consequences of insomnia symptoms among outpatients in primary care practice: EQUINOX international survey. Sleep Med..

[4] Kyle S.D., Beattie L., Spiegelhalder K., Rogers Z., Espie C.A. (2014). Altered emotion perception in insomnia disorder. Sleep.

[5] Spiegelhalder K., Kyle S., Feige B., Prem M., Nissen C., Espie C.A., Riemann D. (2010). The impact of sleep-related attentional bias on polysomnographically measured sleep in primary insomnia. Sleep.

[6] Lin Y.-K., Lin G.-Y., Lee J.-T., Lee M.-S., Tsai C.-K., Hsu Y.-W., Tsai Y.-C., Yang F.-C. (2016). Associations between sleep quality and migraine frequency. Medicine.

[7] Seidel S., Hartl T., Weber M., Matterey S., Pauli A., Riederer F., Gharabaghi M., Wöber-Bingöl C., Wöber C., PAMINA Study Group (2009). Quality of sleep, fatigue and daytime sleepiness in migraine—A controlled study. Cephalalgia.

[8] Rains J.C., Poceta J.S. (2006). Headache and sleep disorders: Review and clinical implications for headache management. Headache J. Head Face Pain.

[9] Stovner L.J., Andree C. (2010). Prevalence of headache in Europe: A review for the Eurolight project. J. Headache Pain.

[10] Haut S.R., Bigal M., Lipton R.B. (2006). Chronic disorders with episodic manifestations: Focus on epilepsy and migraine. Lancet Neurol..

[11] Freedom T., Evans R.W. (2013). Headache and sleep. Headache.

[12] Kelman L., Rains J.C. (2005). Headache and sleep: Examination of sleep patterns and complaints in a large clinical sample of migraineurs. Headache J. Head Face Pain.

[13] Jansson-Fröjmark M., Lindblom K. (2008). A bidirectional relationship between anxiety and depression, and insomnia? A prospective study in the general population. J. Psychosom. Res..

[14] Chu H., Liang C., Lee J., Yeh T., Lee M.-S., Sung Y., Yang F. (2018). Associations between depression/anxiety and headache frequency in migraineurs: A cross-sectional study. Headache J. Head Face Pain.

[15] Leonardi M., Steiner T.J., Scher A.T., Lipton R.B. (2005). The global burden of migraine: Measuring disability in headache disorders with WHO’s Classification of Functioning, Disability and Health (ICF). J. Headache Pain.

[16] Kyle S.D., Morgan K., Espie C.A. (2010). Insomnia and health-related quality of life. Sleep Med. Rev..

[17] Stokes M., Becker W.J., Lipton R.B., Sullivan S.D., Wilcox T.K., Wells L., Manack A., Msc I.P., Gladstone J., Buse D.C. (2011). Cost of health care among patients with chronic and episodic migraine in Canada and the USA: Results from the international burden of migraine study (IBMS). Headache J. Head Face Pain.

[18] Biswal B., Yetkin F.Z., Haughton V.M., Hyde J.S. (1995). Functional connectivity in the motor cortex of resting human brain using echo-planar MRI. Magn. Reson. Med..

[19] Greicius M.D., Krasnow B., Reiss A.L., Menon V. (2003). Functional connectivity in the resting brain: A network analysis of the default mode hypothesis. Proc. Natl. Acad. Sci. USA.

[20] Andrews-Hanna J.R., Saxe R., Yarkoni T. (2014). Contributions of episodic retrieval and mentalizing to autobiographical thought: Evidence from functional neuroimaging, resting-state connectivity, and fMRI meta-analyses. NeuroImage.

[21] Buckner R.L., Andrews-Hanna E.J.R., Schacter D. (2008). The brain’s default network. Ann. N. Y. Acad. Sci..

[22] Heuvel M.V.D., Sporns O. (2013). Network hubs in the human brain. Trends Cogn. Sci..

[23] Buckner R.L., Sepulcre J., Talukdar T., Krienen F.M., Liu H., Hedden T., Andrews-Hanna J.R., Sperling R.A., Johnson K.A. (2009). Cortical hubs revealed by intrinsic functional connectivity: Mapping, assessment of stability, and relation to Alzheimer’s disease. J. Neurosci..

[24] Padmanabhan A., Lynch C., Schaer M., Menon V. (2017). The default mode network in autism. Biol. Psychiatry Cogn. Neurosci. Neuroimaging.

[25] Broyd S.J., Demanuele C., Debener S., Helps S.K., James C.J., Sonuga-Barke E.J. (2009). Default-mode brain dysfunction in mental disorders: A systematic review. Neurosci. Biobehav. Rev..

[26] Zhang J., Su J., Wang M., Zhao Y., Yao Q., Zhang Q., Lu H., Zhang H., Wang S., Li G.-F. (2016). Increased default mode network connectivity and increased regional homogeneity in migraineurs without aura. J. Headache Pain.

[27] Zhou F., Huang S., Gao L., Zhuang Y., Ding S., Gong H. (2016). Temporal regularity of intrinsic cerebral activity in patients with chronic primary insomnia: A brain entropy study using resting-state fMRI. Brain Behav..

[28] Laird A.R., Fox P.M., Eickhoff S.B., Turner J.A., Ray K.L., McKay D.R., Glahn D.C., Beckmann C.F., Smith S.M. (2011). Behavioral Interpretations of Intrinsic Connectivity Networks. J. Cogn. Neurosci..

[29] Andrews-Hanna J.R., Reidler J., Sepulcre J., Poulin R., Buckner R.L. (2010). Functional-anatomic fractionation of the brain’s default network. Neuron.

[30] Damoiseaux J.S., Prater K., Miller B.L., Greicius M.D. (2012). Functional connectivity tracks clinical deterioration in Alzheimer’s disease. Neurobiol. Aging.

[31] Li B., Liu L., Friston K., Shen H., Wang L., Zeng L.-L., Hu D. (2013). A treatment-resistant default mode subnetwork in major depression. Biol. Psychiatry.

[32] Davey C.G., Pujol J., Harrison B. (2016). Mapping the self in the brain’s default mode network. NeuroImage.

[33] Cabeza R., Dolcos F., Graham R., Nyberg L. (2002). Similarities and differences in the neural correlates of episodic memory retrieval and working memory. NeuroImage.

[34] Small D., Gitelman D., Gregory M., Nobre A., Parrish T., Mesulam M.-M. (2003). The posterior cingulate and medial prefrontal cortex mediate the anticipatory allocation of spatial attention. NeuroImage.

[35] Simpson J.R., Drevets W.C., Snyder A.Z., Gusnard D.A., Raichle M.E. (2001). Emotion-induced changes in human medial prefrontal cortex: II. During anticipatory anxiety. Proc. Natl. Acad. Sci. USA.

[36] Wilcox S.L., Veggeberg R., Lemme J., Hodkinson D.J., Scrivani S., Burstein R., Becerra L., Borsook D. (2016). Increased functional activation of limbic brain regions during negative emotional processing in migraine. Front. Hum. Neurosci..

[37] Yan C.-Q., Wang X., Huo J.-W., Zhou P., Li J.-L., Wang Z.-Y., Zhang J., Fu Q.-N., Wang X.-R., Liu C.-Z. (2018). Abnormal global brain functional connectivity in primary insomnia patients: A resting-state functional MRI study. Front. Neurol..

[38] Regier D.A., Kuhl E.A., Kupfer D.J. (2013). The DSM-5: Classification and criteria changes. World Psychiatry.

[39] Bastien C.H., Vallières A., Morin C.M. (2001). Validation of the Insomnia Severity Index as an outcome measure for insomnia research. Sleep Med..

[40] Buysse D.J., Reynolds C.F., Monk T.H., Berman S.R., Kupfer D.J. (1989). The Pittsburgh sleep quality index: A new instrument for psychiatric practice and research. Psychiatry Res..

[41] Headache Classification Committee of the International Headache Society (IHS) (2013). The international classification of headache disorders, 3rd edition (beta version). Cephalalgia.

[42] Zigmond A.S., Snaith R.P. (1983). The hospital anxiety and depression scale. Acta Psychiatr. Scand..

[43] Power J.D., Barnes K.A., Snyder A.Z., Schlaggar B.L., Petersen S.E. (2012). Spurious but systematic correlations in functional connectivity MRI networks arise from subject motion. NeuroImage.

[44] Lefort-Besnard J., Bassett D.S., Smallwood J., Margulies D., Derntl B., Gruber O., Aleman A., Jardri R., Varoquaux G., Thirion B. (2017). Different shades of default mode disturbance in schizophrenia: Subnodal covariance estimation in structure and function. Hum. Brain Mapp..

[45] Friston K.J., Williams S., Howard R., Frackowiak R.S.J., Turner R. (1996). Movement-related effects in fMRI time-series. Magn. Reson. Med..

[46] Murphy K., Fox M.D. (2017). Towards a consensus regarding global signal regression for resting state functional connectivity MRI. NeuroImage.

[47] Yang F.-C., Chou K.-H., Fuh J.-L., Lee P.-L., Lirng J.-F., Lin Y.-Y., Lin C.-P., Wang S.-J. (2015). Altered hypothalamic functional connectivity in cluster headache: A longitudinal resting-state functional MRI study. J. Neurol. Neurosurg. Psychiatry.

[48] Power J.D., Mitra A., Laumann T.O., Snyder A.Z., Schlaggar B.L., Petersen S.E. (2014). Methods to detect, characterize, and remove motion artifact in resting state fMRI. NeuroImage.

[49] Chou K.-H., Lee P.-L., Liang C.-S., Lee J.-T., Kao H.-W., Tsai C.-L., Lin G.-Y., Lin Y.-K., Lin C.-P., Yang F.-C. (2020). Identifying neuroanatomical signatures in insomnia and migraine comorbidity. Sleep.

[50] Ursin H., Eriksen H. (2007). Cognitive activation theory of stress, sensitization, and common health complaints. Ann. N. Y. Acad. Sci..

[51] Tanasescu R., Cottam W., Condon L., Tench C.R., Auer D.P. (2016). Functional reorganisation in chronic pain and neural correlates of pain sensitisation: A coordinate based meta-analysis of 266 cutaneous pain fMRI studies. Neurosci. Biobehav. Rev..

[52] Yang S., Chang M.C. (2019). Chronic pain: Structural and functional changes in brain structures and associated negative affective states. Int. J. Mol. Sci..

[53] Vatthauer K., Craggs J.G., Robinson M., Staud R., Berry R.B., Perlstein W.M., McCrae C.S. (2015). Sleep is associated with task-negative brain activity in fibromyalgia participants with comorbid chronic insomnia. J. Pain Res..

[54] Dai X.-J., Nie X., Shao Y., Liu S.-Y., Li H.-J., Wan A.-L., Nie S., Peng D.-C. (2015). Functional connectivity of paired default mode network subregions in primary insomnia. Neuropsychiatr. Dis. Treat..

[55] Regen W., Kyle S.D., Nissen C., Feige B., Baglioni C., Hennig J., Riemann D., Spiegelhalder K. (2016). Objective sleep disturbances are associated with greater waking resting-state connectivity between the retrosplenial cortex/ hippocampus and various nodes of the default mode network. J. Psychiatry Neurosci..

[56] Xue T., Yuan K., Zhao L., Yu D., Zhao L., Dong T., Cheng P., Von Deneen K.M., Qin W., Tian J. (2012). Intrinsic brain network abnormalities in migraines without aura revealed in resting-state fMRI. PLoS ONE.

[57] Yu D., Yuan K., Zhao L., Zhao L., Dong M., Liu P., Wang G., Liu J., Sun J., Zhou G. (2012). Regional homogeneity abnormalities in patients with interictal migraine without aura: A resting-state study. NMR Biomed..

[58] Raichle M.E., MacLeod A.M., Snyder A.Z., Powers W.J., Gusnard D.A., Shulman G.L. (2001). A default mode of brain function. Proc. Natl. Acad. Sci. USA.

[59] Petrovic P. (2002). Placebo and opioid analgesia—Imaging a shared neuronal network. Science.

[60] Dai X.-J., Nie X., Liu X., Pei L., Jiang J., Peng D.-C., Gong H.-H., Zeng X.-J., Wáng Y.-X.J., Zhan Y. (2016). Gender differences in regional brain activity in patients with chronic primary insomnia: Evidence from a resting-state fMRI study. J. Clin. Sleep Med..

[61] Dai X.-J., Peng D.-C., Gong H.-H., Wan A.-L., Nie X., Li H.-J., Wang Y.-X. (2014). Altered intrinsic regional brain spontaneous activity and subjective sleep quality in patients with chronic primary insomnia: A resting-state fMRI study. Neuropsychiatr. Dis. Treat..

[62] Li Y., Wang E., Zhang H., Dou S., Liu L., Tong L., Lei Y., Wang M., Xu J., Shi D. (2014). Functional connectivity changes between parietal and prefrontal cortices in primary insomnia patients: Evidence from resting-state fMRI. Eur. J. Med. Res..

[63] Cavanna A.E., Trimble M.R. (2006). The precuneus: A review of its functional anatomy and behavioural correlates. Brain.

[64] Goffaux P., Girard-Tremblay L., Marchand S., Daigle K., Whittingstall K. (2014). Individual differences in pain sensitivity vary as a function of precuneus reactivity. Brain Topogr..

[65] Seifert F., Maihöfner C. (2011). Functional and structural imaging of pain-induced neuroplasticity. Curr. Opin. Anaesthesiol..

[66] Georgopoulos A.P. (2000). Neural aspects of cognitive motor control. Curr. Opin. Neurobiol..

[67] Valfrè W., Rainero I., Bergui M., Pinessi L. (2007). Voxel-based morphometry reveals gray matter abnormalities in migraine. Headache J. Head Face Pain.

[68] Tamura Y., Okabe S., Ohnishi T., Saito D.N., Arai N., Mochio S., Inoue K., Ugawa Y. (2004). Effects of 1-Hz repetitive transcranial magnetic stimulation on acute pain induced by capsaicin. Pain.

[69] Wang T., Li S., Jiang G., Lin C., Li M., Ma X., Zhan W., Fang J., Li L., Li C. (2016). Regional homogeneity changes in patients with primary insomnia. Eur. Radiol..

[70] Huang Z., Liang P., Jia X., Zhan S., Li N., Ding Y., Lu J., Wang Y., Li K. (2012). Abnormal amygdala connectivity in patients with primary insomnia: Evidence from resting state fMRI. Eur. J. Radiol..

[71] Mehnert J., Schulte L., Timmann D., May A. (2017). Activity and connectivity of the cerebellum in trigeminal nociception. NeuroImage.

[72] Goadsby P., Holland P., Martins-Oliveira M., Hoffmann J., Schankin C., Akerman S. (2017). Pathophysiology of migraine: A disorder of sensory processing. Physiol. Rev..

[73] Jiang G., Li C., Ma X., Dong M., Yin Y., Hua K., Li M., Li C., Zhan W., Li C. (2016). Abnormal spontaneous regional brain activity in primary insomnia: A resting-state functional magnetic resonance imaging study. Neuropsychiatr. Dis. Treat..

[74] Li C., Dong M., Yin Y., Hua K., Fu S., Jiang G. (2018). Aberrant effective connectivity of the right anterior insula in primary insomnia. Front. Neurol..

[75] Zhang J., Su J., Wang M., Zhao Y., Zhang Q.-T., Yao Q., Lu H., Zhang H., Li G.-F., Wu Y.-L. (2017). The sensorimotor network dysfunction in migraineurs without aura: A resting-state fMRI study. J. Neurol..

[76] Iliff J.J., Wang M., Liao Y., Plogg B.A., Peng W., Gundersen G.A., Benveniste H., Vates G.E., Deane R., Goldman S. (2012). A paravascular pathway facilitates CSF flow through the brain parenchyma and the clearance of interstitial solutes, including amyloid β. Sci. Transl. Med..

[77] Christensen J., Yamakawa G.R., Shultz S.R., Mychasiuk R. (2021). Is the glymphatic system the missing link between sleep impairments and neurological disorders? Examining the implications and uncertainties. Prog. Neurobiol..

[78] Schain A.J., Melo-Carrillo A., Strassman A.M., Burstein R. (2017). Cortical spreading depression closes paravascular space and impairs glymphatic flow: Implications for migraine headache. J. Neurosci..

[79] Roh J.H., Huang Y., Bero A.W., Kasten T., Stewart F.R., Bateman R., Holtzman D.M. (2012). Disruption of the sleep-wake cycle and diurnal fluctuation of -amyloid in mice with alzheimer’s disease pathology. Sci. Transl. Med..

[80] Ringstad G., Vatnehol S.A.S., Eide P.K. (2017). Glymphatic MRI in idiopathic normal pressure hydrocephalus. Brain.

[81] Ringstad G., Valnes L.M., Dale A.M., Pripp A.H., Vatnehol S.A.S., Emblem K., Mardal K.-A., Eide P.K. (2018). Brain-wide glymphatic enhancement and clearance in humans assessed with MRI. JCI Insight.

[82] Mueller S., Wang D., Fox M.D., Yeo B.T., Sepulcre J., Sabuncu M., Shafee R., Lu J., Liu H. (2013). Individual variability in functional connectivity architecture of the human brain. Neuron.

[83] Wang D., Buckner R.L., Fox M.D., Holt D.J., Holmes A.J., Stoecklein S., Langs G., Pan R., Qian T., Li K. (2015). Parcellating cortical functional networks in individuals. Nat. Neurosci..

[84] Kushida C.A., Littner M.R., Morgenthaler T., Alessi C.A., Bailey D., Coleman J., Friedman L., Hirshkowitz M., Kapen S., Kramer M. (2005). Practice parameters for the indications for polysomnography and related procedures: An update for 2005. Sleep.

