# Supplementary material for: Shared Patterns of Brain Functional Connectivity for the Comorbidity between Migraine and Insomnia
Source: Biomedicines. 2021 Oct 9;9(10):1420. doi: 10.3390/biomedicines9101420 (PMC8533078; doi:10.3390/biomedicines9101420)
Supplement: Supplementary file 1 [file biomedicines-09-01420-s001.zip › biomedicines-1393961-SI.pdf]

Supplementary Figure Legends

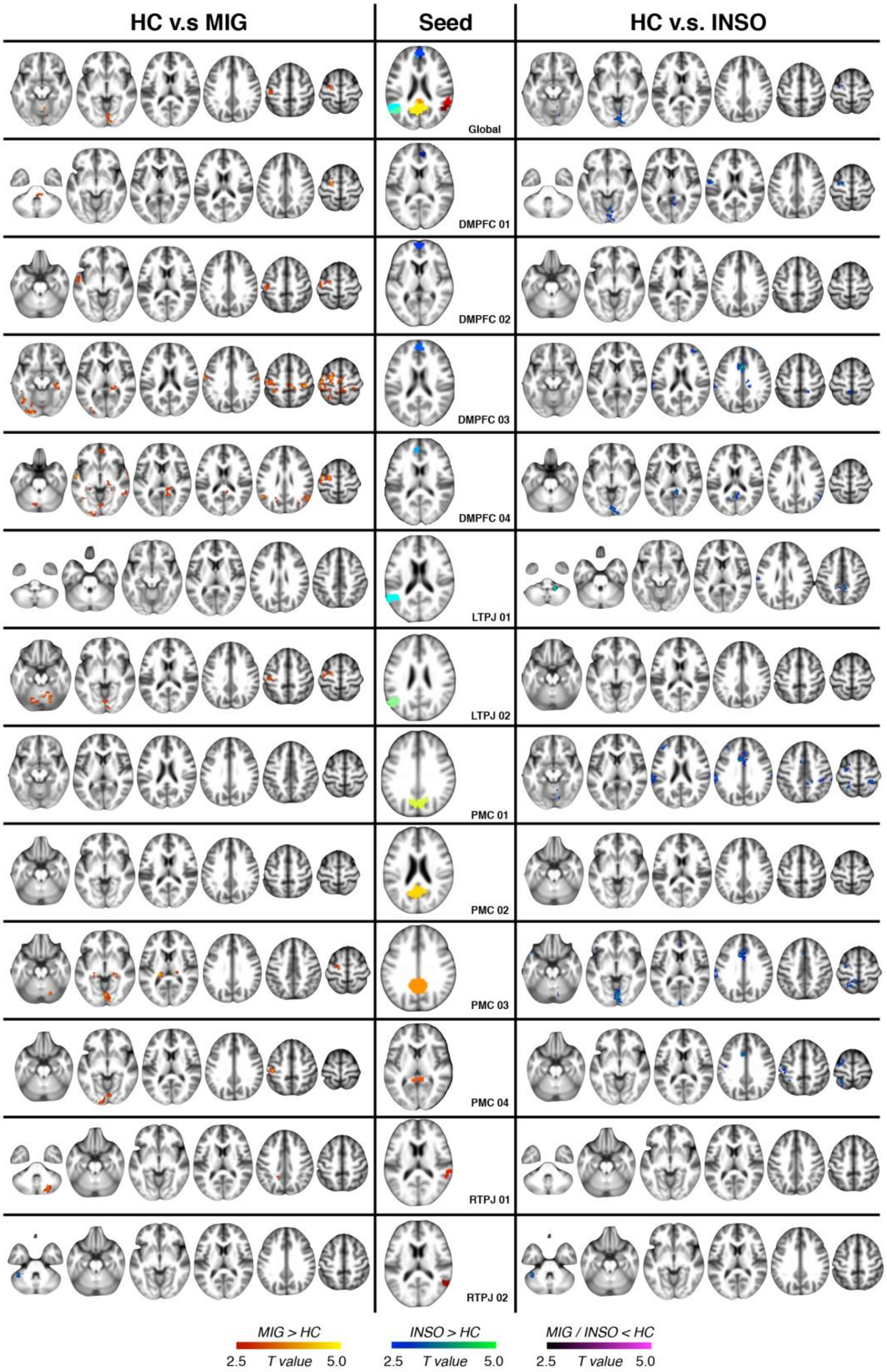

Supplementary Figure S1. The detailed FC differences of the global DMN and subnodal DMN between healthy controls and clinical groups (migraine and insomnia). Red-Yellow regions show significantly increased FC in patients with migraine. Blue-Green regions show significantly increased FC in patients with insomnia. Purple regions show significantly increased FC in healthy controls. Abbreviations: DMPFC, dorsomedial prefrontal cortex; FC, functional connectivity; HC, healthy controls; INSO, insomnia; LTPJ, left temporoparietal junctions; MIG, migraine; PMC, posteromedial cortex; RTPJ, right temporoparietal junctions.

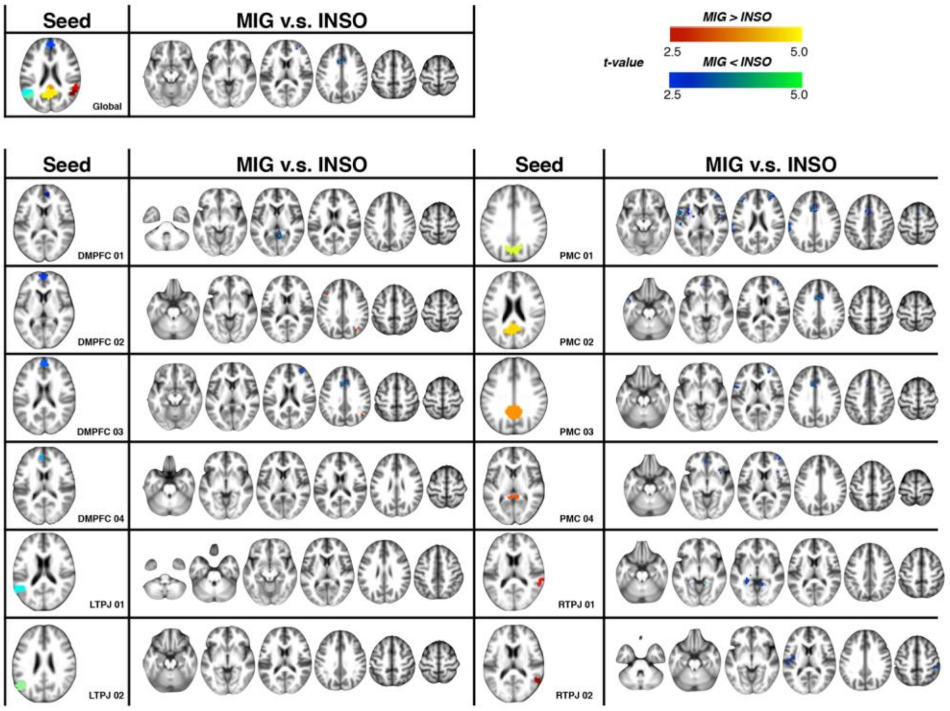

Supplementary Figure S2. The detailed FC differences of the global DMN and subnodal DMN between patients with migraine and patients with insomnia. Red-Yellow regions reflect significantly increased FC in patients with migraine. Blue-Green regions show significantly increased FC in patients with insomnia. Abbreviations: DMPFC, dorsomedial prefrontal cortex; FC, functional connectivity; INSO, insomnia; LTPJ, left temporoparietal junctions; MIG, migraine; PMC, posteromedial cortex; RTPJ, right temporoparietal junctions.

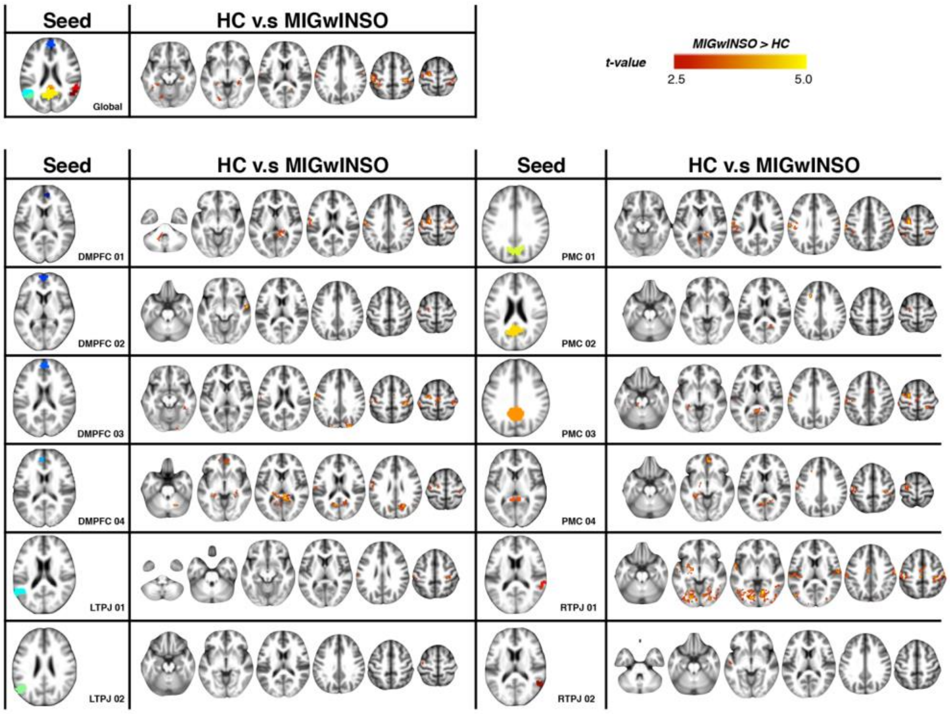

Supplementary Figure S3. The detailed FC differences of the global DMN and subnodal DMN between healthy controls and patients with comorbid migraine and insomnia. Hot (red-yellow) regions represent significantly increased FC in patients with comorbid migraine and insomnia. Abbreviations: DMPFC, dorsomedial prefrontal cortex; FC, functional connectivity; HC, healthy controls; LTPJ, left temporoparietal junctions; MIGwINSO, migraine with insomnia; PMC, posteromedial cortex; RTPJ, right temporoparietal junctions.

Supplementary Tables

Supplementary Table S1. Anatomical regions with significant FC changes in migraine patients, insomnia patients compared with healthy controls.

MNI coordinates			Cluster	Maximum T	Anatomical region
x	y	z	size	value	
Global DMN					
HC < MIG					
-26	-18	76	182	4.80	Lt. Precentral Gyrus
6	-62	-10	201	4.53	Rt. Cerebellum V
-48	-21	59	131	3.90	Lt. Postcentral Gyrus
HC < INSO					
-24	-10	72	136	4.13	Lt. Precentral Gyrus
6	-64	-8	248	3.95	Rt. Cerebellum V
MIG < INSO					
-6	13	35	170	4.45	Lt. Cingulate Gyrus, anterior division
34	59	21	120	3.58	Rt. Frontal Pole
DMPFC 01					
HC < MIG					
11	-36	-36	123	4.55	Brain stem
-24	-13	73	169	3.77	Lt. Precentral Gyrus
HC < INSO					
2	-84	-6	303	3.98	Rt. Lingual Gyrus
8	-43	9	157	3.96	Rt. Cingulate Gyrus

-60	-10	16	156	3.26	Lt. Postcentral Gyrus
-30	-11	68	111	3.60	Lt. Precentral Gyrus
MIG < INSO					
6	-48	4	197	4.79	Rt. Cingulate Gyrus, posterior division
DMPFC 02					
HC < MIG					
-25	-14	74	155	4.35	Lt. Precentral Gyrus
-56	-1	-6	111	4.13	Lt. Superior Temporal Gyrus, anterior division
-49	-23	53	155	3.76	Lt. Postcentral Gyrus
MIG > INSO					
-36	22	32	114	4.37	Lt. Middle Frontal Gyrus
40	-61	47	168	4.29	Rt. Lateral Occipital Cortex, superior division
DMPFC 03					
HC < MIG					
4	-28	56	727	3.57	Rt. Precentral Gyrus
-22	-12	64	1667	5.03	Lt. Precentral Gyrus
-36	-68	-2	757	4.90	Lt. Lateral Occipital Cortex, inferior division
27	-39	-4	331	4.03	Rt. Lingual Gyrus
8	-82	-6	224	3.70	Rt. Lingual Gyrus
HC < INSO					

-2	12	36	346	4.95	Lt. Cingulate Gyrus, anterior division
16	-30	42	213	4.21	Rt. Precentral Gyrus
-4	-32	68	136	3.37	Lt. Precentral Gyrus
-66	-34	25	146	3.68	Lt. Supramarginal Gyrus, anterior division
31	49	20	145	3.59	Rt. Frontal Pole
MIG > INSO					
42	-62	41	147	3.42	Rt. Lateral Occipital Cortex, superior division
MIG < INSO					
4	20	38	236	4.72	Rt. Paracingulate Gyrus
30	46	16	148	4.02	Rt. Frontal Pole
DMPFC 04					
HC < MIG					
12	-37	7	570	4.27	Rt. Cingulate Gyrus, posterior division
-56	-8	-4	206	4.82	Lt. Superior Temporal Gyrus, anterior division
-25	-15	74	381	4.81	Lt. Precentral Gyrus
-6	-70	-42	680	4.39	Lt. Cerebellum VIIb
-44	-66	-12	125	4.35	Lt. Lateral Occipital Cortex, inferior division
-48	-54	30	115	4.29	Lt. Angular Gyrus
-28	-30	-12	246	3.48	Lt. Hippocampus

55	-5	-9	307	4.10	Rt. Superior Temporal Gyrus, anterior division
5	49	-9	125	4.06	Rt. Frontal Medial Cortex
54	-59	32	379	3.89	Rt. Lateral Occipital Cortex, superior division
-20	-62	50	161	3.85	Lt. Lateral Occipital Cortex, superior division
60	-36	-9	228	3.74	Rt. Middle Temporal Gyrus, posterior division
HC < INSO					
18	-93	-1	213	4.45	Rt. Occipital Pole
8	-46	18	248	4.37	Rt. Cingulate Gyrus, posterior division
57	-60	37	156	3.59	Rt. Lateral Occipital Cortex, superior division
PMC 01					
HC < INSO					
4	-64	-10	122	5.22	Rt. Cerebellum V
-60	-30	36	439	4.55	Lt. Supramarginal Gyrus, anterior division
-10	14	34	464	4.52	Lt. Cingulate Gyrus, anterior division
-39	39	23	144	4.37	Lt. Frontal Pole
-26	-50	68	178	4.29	Lt. Superior Parietal Lobule

66	-22	33	193	4.19	Rt. Supramarginal Gyrus, anterior division
-13	5	58	157	4.12	Lt. Superior Frontal Gyrus
44	-34	42	266	3.91	Rt. Supramarginal Gyrus, anterior division
18	-36	44	120	3.87	Rt. Precentral Gyrus
MIG < INSO					
-7	14	34	619	4.95	Lt. Cingulate Gyrus, anterior division
-38	40	29	217	4.84	Lt. Frontal Pole
-54	8	6	257	4.57	Lt. Precentral Gyrus
-68	-26	20	489	4.13	Lt. Supramarginal Gyrus, anterior division
-30	8	10	207	4.00	Lt. Insular Cortex
37	51	20	294	3.91	Rt. Frontal Pole
50	-12	12	137	3.76	Rt. Central Opercular Cortex
54	-60	-10	112	3.71	Rt. Inferior Temporal Gyrus
PMC 02					
MIG < INSO					
-5	16	33	204	4.65	Lt. Cingulate Gyrus, anterior division
-50	14	-12	133	3.90	Lt. Temporal Pole
-5	44	-8	147	3.78	Lt. Paracingulate Gyrus
38	52	18	150	3.63	Rt. Frontal Pole
PMC 03					

HC < MIG					
-18	-29	9	296	4.94	Lt. Thalamus
6	-62	-11	556	4.60	Rt. Cerebellum V
22	-22	6	257	4.50	Rt. Thalamus
-31	-11	72	129	4.39	Lt. Precentral Gyrus
HC < INSO					
4	-64	-10	761	4.92	Rt. Cerebellum V
-10	14	34	483	4.37	Lt. Cingulate Gyrus, anterior division
-48	22	-26	180	4.19	Lt. Temporal Pole
-63	-33	38	279	4.08	Lt. Supramarginal Gyrus, anterior division
-26	-10	74	119	4.08	Lt. Precentral Gyrus
4	12	70	159	3.44	Rt. Superior Frontal Gyrus
-16	-46	67	272	3.82	Lt. Postcentral Gyrus
MIG < INSO					
-4	14	36	309	4.97	Lt. Cingulate Gyrus, anterior division
-58	14	4	132	4.44	Lt. Inferior Frontal Gyrus, pars opercularis
34	46	22	232	3.79	Rt. Frontal Pole
PMC 04					
HC < MIG					
8	-90	-2	148	4.22	Rt. Occipital Pole
-45	-25	54	176	4.10	Lt. Postcentral Gyrus

-13	-97	-5	133	3.89	Lt. Occipital Pole
HC < INSO					
-10	14	34	124	4.21	Lt. Cingulate Gyrus, anterior division
-32	-12	66	142	3.97	Lt. Precentral Gyrus
-17	-55	65	203	3.88	Lt. Superior Parietal Lobule
-48	-22	42	299	3.73	Lt. Postcentral Gyrus
MIG < INSO					
34	20	-10	163	4.59	Rt. Frontal Orbital Cortex
39	53	19	210	3.85	Rt. Frontal Pole
-4	46	-8	165	3.70	Lt. Paracingulate Gyrus
LTPJ 01					
HC < INSO					
18	-44	-46	126	5.39	Rt. Cerebellum X
6	-42	46	194	4.45	Rt. Precuneous Cortex
-66	-24	32	129	4.38	Lt. Supramarginal Gyrus, anterior division
LTPJ 02					
HC < MIG					
-38	-16	62	251	4.14	Lt. Precentral Gyrus
22	-70	-20	130	4.11	Rt. Cerebellum VI
6	-62	-22	111	4.11	Rt. Cerebellum V
-4	-78	-12	249	3.75	Cerebellum Vermis VI
RTPJ 01					

HC < MIG					
-20	-52	30	112	4.47	Lt. Precuneous Cortex
26	-68	-40	180	4.31	Rt. Cerebellum Crus II
MIG < INSO					
12	-48	-6	162	4.16	Rt. Cerebellum V
-27	-39	-4	198	3.5	Lt. Hippocampus
RTPJ 02					
HC < INSO					
-42	-48	-34	117	4.08	Lt. Cerebellum Crus I
MIG < INSO					
44	-34	56	189	4.16	Rt. Postcentral Gyrus
-53	-19	11	142	3.71	Lt. Central Opercular Cortex

Peak of group differences in FC magnitude of the global DMN and subnodal DMN with a threshold of FWE-corrected p-value < 0.05.

Abbreviations: DMN, default mode network; DMPFC, dorsomedial prefrontal cortex; FC, functional connectivity; HC, healthy controls; INSO, insomnia; Lt, left; LTPJ, left temporoparietal junctions; MIG, migraine; MNI, Montreal Neurological Institute; PMC, posteromedial cortex; Rt, right; RTPJ, right temporoparietal junctions.

Supplementary Table S2. Anatomical regions with significant FC changes in patients with comorbid migraine and insomnia compared with healthy controls.

MNI coordinates			Cluster	Maximum T	Anatomical region
x	y	z	size	value	
Global DMN					
HC < MIGwINSO					
-22	-16	76	391	5.61	Lt. Precentral gyrus
36	-28	54	292	5.09	Rt. Postcentral gyrus
10	-39	7	235	4.79	Rt. Cingulate gyrus
-49	-26	58	911	4.67	Lt. Postcentral gyrus
-18	-36	4	238	4.46	Lt. Thalamus
28	-35	-5	144	4.38	Rt. Hippocampus
-18	-66	-18	177	3.31	Lt. Cerebellum VI
61	-15	42	130	3.99	Rt. Postcentral gyrus
DMPFC 01					
HC < MIGwINSO					
-22	-14	76	1371	5.96	Lt. Precentral gyrus
-6	-40	-36	123	4.95	Lt. brain stem
28	-30	56	222	4.29	Rt. Postcentral Gyrus
9	-44	8	143	4.27	Rt. Cingulate Gyrus, posterior division
7	-17	58	248	4.13	Rt. Precentral Gyrus
18	-24	64	112	4.06	Rt. Precentral Gyrus
44	-18	40	180	3.89	Rt. Postcentral Gyrus

DMPFC 02					
HC < MIGwINSO					
57	-4	-12	124	4.95	Rt. Superior Temporal Gyrus, anterior division
-26	-20	74	138	4.57	Lt. Precentral Gyrus
DMPFC 03					
HC < MIGwINSO					
36	-30	54	210	5.69	Rt. Postcentral Gyrus
-64	-10	36	486	5.29	Lt. Postcentral Gyrus
4	-14	68	363	4.18	Rt. Precentral Gyrus
24	-88	36	495	4.89	Rt. Occipital Pole
-32	-10	62	118	4.56	Lt. Precentral Gyrus
-20	-84	34	114	4.21	Lt. Lateral Occipital Cortex, superior division
32	-40	-10	111	3.54	Rt. Lingual Gyrus
24	-86	-7	140	3.45	Rt. Occipital Fusiform Gyrus
DMPFC 04					
HC < MIGwINSO					
9	-41	7	1664	4.67	Rt. Cingulate Gyrus, posterior division
58	-4	-14	306	5.49	Rt. Superior Temporal Gyrus, anterior division
19	-62	-29	253	5.09	Rt. Cerebellum VI

38	-61	39	465	5.05	Rt. Lateral Occipital Cortex, superior division
-55	-18	43	466	4.72	Lt. Postcentral Gyrus
-14	-51	-39	160	4.29	Lt. Cerebellum IX
-27	-17	75	141	4.32	Lt. Precentral Gyrus
27	-38	-3	258	4.06	Rt. Hippocampus
39	-26	54	145	4.13	Rt. Postcentral Gyrus
-6	45	-8	254	3.87	Lt. Paracingulate Gyrus
PMC 01					
HC < MIGwINSO					
-22	-9	64	345	4.93	Lt. Precentral Gyrus
-60	-30	38	1016	4.89	Lt. Supramarginal Gyrus, anterior division
64	-18	40	377	4.37	Rt. Postcentral Gyrus
15	-39	4	167	4.00	Rt. Cingulate Gyrus, posterior division
24	-38	64	130	3.93	Rt. Postcentral Gyrus
PMC 02					
HC < MIGwINSO					
14	-62	22	260	4.90	Rt. Precuneous Cortex
-26	24	36	123	4.79	Lt. Middle Frontal Gyrus
-21	-14	62	134	4.05	Lt. Precentral Gyrus
PMC 03					
HC < MIGwINSO					

14	-46	6	411	4.90	Rt. Cingulate Gyrus, posterior division
-25	-14	74	340	4.88	Lt. Precentral Gyrus
-19	-37	-29	146	4.87	Lt. Cerebellum I-IV
-64	-19	33	324	4.79	Lt. Postcentral Gyrus
-52	-22	60	300	4.31	Lt. Postcentral Gyrus
-37	-36	-19	207	4.26	Lt. Temporal Fusiform Cortex, posterior division
15	-37	69	163	4.01	Rt. Postcentral Gyrus
-4	-22	62	180	3.82	Lt. Precentral Gyrus
44	-34	54	124	3.81	Rt. Postcentral Gyrus
PMC 04					
HC < MIGwINSO					
-11	-54	20	565	5.00	Lt. Precuneous Cortex
-57	-22	44	484	4.83	Lt. Postcentral Gyrus
-23	-15	75	230	4.63	Lt. Precentral Gyrus
4	50	-6	213	4.57	Rt. Paracingulate Gyrus
-28	22	46	145	4.56	Lt. Middle Frontal Gyrus
-4	-60	-48	177	4.51	Lt. Cerebellum IX
-28	-36	-6	248	4.12	Lt. Hippocampus
34	-26	52	123	4.01	Rt. Postcentral Gyrus
LTPJ 01					
HC < MIGwINSO					
-61	-22	44	285	4.18	Lt. Postcentral Gyrus

32	-29	55	123	3.80	Rt. Postcentral Gyrus
LTPJ 02					
HC < MIGwINSO					
-36	-10	63	116	4.77	Lt. Precentral Gyrus
RTPJ 01					
HC < MIGwINSO					
-52	-62	4	1306	6.25	Lt. Middle Temporal Gyrus, temporooccipital part
14	-69	-2	926	5.56	Rt. Lingual Gyrus
-60	-24	46	576	5.17	Lt. Postcentral Gyrus
-4	-26	56	378	5.03	Lt. Precentral Gyrus
64	-14	36	1196	5.02	Rt. Postcentral Gyrus
30	-56	-60	262	4.87	Rt. Cerebellum VIIIa
-22	-82	30	241	4.65	Lt. Lateral Occipital Cortex, superior division
-55	-5	-3	161	4.51	Lt. Superior Temporal Gyrus, anterior division
22	-84	31	151	4.44	Rt. Lateral Occipital Cortex, superior division
-67	-22	15	293	4.20	Lt. Postcentral Gyrus
-24	-12	62	212	4.14	Lt. Precentral Gyrus
20	-46	75	129	4.12	Rt. Superior Parietal Lobule
RTPJ 02					

HC < MIGwINSO					
-54	-22	-10	113	3.91	Lt. Middle Temporal Gyrus, posterior division

Peak of group differences in FC magnitude of the global DMN and subnodal DMN with a threshold of FWE-corrected p-value < 0.05.

Abbreviations: DMN, default mode network; DMPFC, dorsomedial prefrontal cortex; FC, functional connectivity; HC, healthy controls; Lt, left; LTPJ, left temporoparietal junctions; MIGwINSO, migraine with insomnia; MNI, Montreal Neurological Institute; PMC, posteromedial cortex; Rt, right; RTPJ, right temporoparietal junctions.
